# Supplementary material for: An open-source k-mer based machine learning tool for fast and accurate subtyping of HIV-1 genomes
Source: PLoS One. 2018 Nov 14;13(11):e0206409. doi: 10.1371/journal.pone.0206409 (PMC6235296; doi:10.1371/journal.pone.0206409)
Supplement: S1 Appendix — (PDF) [file pone.0206409.s001.pdf]

Appendix 1

Instructions for reproduction of the experiments using Kameris

First, visit `https://github.com/stephensolis/kameris` and follow the instructions for installing Kameris. It is highly recommended you also work through the demo instructions on the same page, to understand how the software works.

1 10-fold cross-validation on the full set of full-length HIV-1 genomes

Run `kameris run-job https://raw.githubusercontent.com/stephensolis/kameris-experiments/master/experiments/hiv1/lanl-whole.yml https://raw.githubusercontent.com/stephensolis/kameris/master/demo/settings.yml`

2 10-fold cross-validation on the full set of HIV-1 pol genes

Run `kameris run-job https://raw.githubusercontent.com/stephensolis/kameris-experiments/master/experiments/hiv1/lanl-pol.yml https://raw.githubusercontent.com/stephensolis/kameris/master/demo/settings.yml`

3 Classification of the HIV-1 benchmark dataset

1. Run `kameris run-job https://raw.githubusercontent.com/stephensolis/kameris-experiments/master/experiments/hiv1/lanl-reference-model.yml https://raw.githubusercontent.com/stephensolis/kameris/master/demo/settings.yml` to train the model.

2. Download `https://drive.google.com/uc?export=download&id=0B7OX388vZjTva1NNYXh5WEM2Z28` and extract the `mixed-polfragments` folder.

3. Run `kameris classify output/lanl-reference-model/subtype-k=6/model_linear-svm.mm-model "path to the mixed-polfragments folder"`

4. Compare the output stored in `results.json` with the ground-truth subtypes from `https://raw.githubusercontent.com/stephensolis/kameris-experiments/master/metadata/hiv1-mixed-polfragments.json`.

4 10-fold cross-validation on the synthetic-vs-natural HIV-1 pol genes

Run `kameris run-job` `https://raw.githubusercontent.com/stephensolis/kameris-experiments/master/experiments/hiv1/real-vs-synthetic.yml` `https://raw.githubusercontent.com/stephensolis/kameris/master/demo/settings.yml`

5 Classification of randomly-generated sequence

1. Follow steps 1-2 from section 3.
2. Generate a random sequence, for example using `http://www.faculty.ucr.edu/~mmaduro/random.htm`, save it to a file, and put the file in a new folder by itself.
3. Run `kameris classify output/lanl-reference-model/subtype-k=6/model_linear-svm.mm-model "path to the folder you created"`

6 10-fold cross-validation on the set of whole dengue virus genomes

Run `kameris run-job` `https://raw.githubusercontent.com/stephensolis/kameris-experiments/master/experiments/dengue/ncbi-whole.yml` `https://raw.githubusercontent.com/stephensolis/kameris/master/demo/settings.yml`

7 10-fold cross-validation on the set of whole hepatitis B genomes

Run `kameris run-job` `https://raw.githubusercontent.com/stephensolis/kameris-experiments/master/experiments/hepatitis/hbv-whole.yml` `https://raw.githubusercontent.com/stephensolis/kameris/master/demo/settings.yml`

8 10-fold cross-validation on the set of whole hepatitis C genomes

Run `kameris run-job` `https://raw.githubusercontent.com/stephensolis/kameris-experiments/master/experiments/hepatitis/hcv-whole.yml` `https://raw.githubusercontent.com/stephensolis/kameris/master/demo/settings.yml`

9 10-fold cross-validation on the set of whole influenza A genomes

Run `kameris run-job` `https://raw.githubusercontent.com/stephensolis/kameris-experiments/master/experiments/flu/ncbi-whole.yml` `https://raw.githubusercontent.com/stephensolis/kameris/master/demo/settings.yml`
