# Supplementary material for: An open-source k-mer based machine learning tool for fast and accurate subtyping of HIV-1 genomes
Source: PLoS One. 2018 Nov 14;13(11):e0206409. doi: 10.1371/journal.pone.0206409 (PMC6235296; doi:10.1371/journal.pone.0206409)
Supplement: S2 Appendix — (PDF) [file pone.0206409.s002.pdf]

Appendix 2

List of subtypes of viral species from datasets used

1 Whole HIV-1 genomes

Subtype	Recombinant?
01B	Yes
01BC	Yes
01_AE	Yes
02A1	Yes
02_AG	Yes
07_BC	Yes
08_BC	Yes
11_cpx	Yes
35_AD	Yes
A1	No
A1C	Yes
A1CD	Yes
A1D	Yes
A6	No
B	No
BC	Yes
BF	Yes
BF1	Yes
C	No
CD	Yes
D	No
F1	No
G	No
O	No
U	No

2 Full set of HIV-1 pol genes

Subtype	Recombinant?
0107	Yes
01B	Yes
01BC	Yes
01_AE	Yes
02A1	Yes
02_AG	Yes
07_BC	Yes
08_BC	Yes
11_cpx	Yes
35_AD	Yes
A1	No
A1C	Yes
A1CD	Yes
A1D	Yes
A6	No
B	No
BC	Yes
BF	Yes
BF1	Yes
C	No
CD	Yes
D	No
F1	No
G	No
O	No
U	No

3 HIV-1 pol genes from the 2010 LANL Web alignment

Subtype	Recombinant?
01B	Yes
01_AE	Yes
02_AG	Yes
A1	No
A1C	Yes
A1D	Yes
B	No
BC	Yes
BF	Yes
BF1	Yes
C	No
D	No
F1	No
G	No
O	No

4 Whole dengue virus genomes

Subtype
1
2
3
4

5 Whole hepatitis B genomes

Subtype	Recombinant?
A	No
B	No
C	No
D	No
E	No
F	No
G	No
H	No
RF-BC	Yes
RF-CB	Yes
RF-DC	Yes
RF-DE	Yes

6 Whole hepatitis C genomes

Subtype
1a
1b
2a
2b
3a
6a

7 Whole influenza A genomes

Subtype	Subtype
H1N1	H6N6
H1N2	H6N8
H1N3	H7N1
H1N6	H7N2
H1N9	H7N3
H2N1	H7N4
H2N2	H7N6
H2N3	H7N7
H2N7	H7N9
H2N9	H8N4
H3N1	H9N2
H3N2	H10N1
H3N3	H10N3
H3N6	H10N4
H3N8	H10N5
H4N2	H10N6
H4N6	H10N7
H4N8	H10N8
H4N9	H11N1
H5N1	H11N2
H5N2	H11N3
H5N3	H11N9
H5N5	H12N5
H5N6	H13N2
H5N8	H13N6
H6N1	H13N8
H6N2	H16N3
H6N5	mixed
